# An M.D. Self-Qualified

**Published:** 1892-06

**Authors:** 


### An M.D. Self-Qualified.

There is a sharp-witted dentist in New York City who is liable to get into legal trouble through his smartness. His name is reported as Alexander Walter, he claims to have been in dental practice twenty years, and he writes "M.D." after his name. He was arrested on four charges that he was engaged in the practice of medicine without license or diploma. In his statement he entered the plea that the letters M.D. were used to show that he was a "Mechanical Dentist," and that he had never represented himself as a doctor of medicine. His trial is still on.

